# Increased risk of Parkinson’s disease among patients with age-related macular degeneration

**DOI:** 10.1186/s12886-021-02196-8

**Published:** 2021-12-09

**Authors:** Po-Yu Jay Chen, Lei Wan, Jung-Nien Lai, Chih Sheng Chen, Jamie Jiin-Yi Chen, Wu Ming Yen, Lu-Ting Chiu, Kai-Chieh Hu, Peng-Tai Tien, Hui-Ju Lin

**Affiliations:** 1 Eye Center and Department of Molecular Genetics, China Medical University Hospital, Taichung, Taiwan; 2 School of Chinese Medicine, China Medical University, Taichung, Taiwan; 3 Department of Medical Laboratory Science and Biotechnology, Asia University, Taichung, Taiwan; 4 Department of Obstetrics and Gynecology, China Medical University Hospital, Taichung, Taiwan; 5 Division of Chinese Medicine, Asia University Hospital, Taichung, Taiwan; 6 Management Office for Health Data, China Medical University Hospital, Taichung, Taiwan; 7 Graduate Institute of Clinical Medical Science, College of Medicine, China Medical University, Taichung, Taiwan; 8 School of Medicine, College of Medicine, China Medical University, Taichung, Taiwan; 9 Department of Ophthalmology and Department of Molecular Genetics, China Medical University Hospital, Taichung, Taiwan

**Keywords:** Parkinson’s disease (PD), Age-related macular degeneration (AMD), Retinal inflammation

## Abstract

**Background:**

This study aimed to investigate the risk of Parkinson’s disease (PD) among patients with age-related macular degeneration (AMD) and its association with confounding comorbidities.

**Methods:**

A population-based retrospective cohort study was conducted using Longitudinal Health Insurance Database 2000 (LHID2000). We established AMD and non-AMD cohorts from January 1, 2000 to December 31, 2012 to determine the diagnosis of PD. A total of 20,848 patients were enrolled, with 10,424 AMD patients and 10,424 controls matched for age, sex, and index year at a 1:1 ratio. The follow-up period was from the index date of AMD diagnosis to the diagnosis of PD, death, withdrawal from the insurance program, or end of 2013. Multivariable Cox regression analysis was performed to examine the hazard ratio (HR) and 95% confidence interval (CI) for the risk of PD between the AMD and non-AMD cohorts.

**Result:**

After adjusting for potential confounders, there was a higher risk of developing PD in the AMD cohort than in the non-AMD cohort (adjusted HR = 1.35, 95% CI = 1.16–1.58). A significant association could be observed in both female (aHR = 1.42, 95% CI = 1.13–1.80) and male (aHR = 1.28, 95% CI = 1.05–1.57) patients, aged more than 60 years (60–69: aHR = 1.51, 95% CI = 1.09–2.09, 70–79: aHR = 1.30, 95% CI = 1.05–1.60; 80–100: aHR = 1.40, 95% CI = 1.01–1.95), and with more than one comorbidity (aHR = 1.40, 95% CI = 1.20–1.64). A significant association between increased risk of PD and AMD was observed among patients with comorbidities of osteoporosis (aHR = 1.68, 95% CI = 1.22–2.33), diabetes (aHR = 1.41, 95% CI = 1.12–1.78) and hypertension (aHR = 1.36, 95% CI = 1.15–1.62) and medications of statin (aHR = 1.42, 95% CI = 1.19–1.69) and calcium channel blocker (CCB) (aHR = 1.32, 95% CI = 1.11–1.58). The cumulative incidence of PD was significantly higher over the 12-year follow-up period in AMD cohort (log-rank test, *p* < 0.001).

**Conclusions:**

Patients with AMD may exhibit a higher risk of PD than those without AMD.

## Introduction

Age-related macular degeneration (AMD) is one of the causes of blindness among the elderly, affecting the central area of the retina, causing thinning of the retinal pigment epithelium (RPE) and retinal nerve fiber layer (RNFL) [1]. Typically, AMD affects people older than 55 years [2]. Symptoms of AMD can range from asymptomatic to visual distortion and loss of central vision. Additionally, AMD is classified as neovascular or non-neovascular based on the presentation of growing abnormal blood vessels from either choroidal or retinal circulation [3]. Among those with non-neovascular AMD, the annual risk of developing neovascular AMD is about 1 to 4.7% [3].

On the other hand, PD is a central neural degenerative disorder among people aged around 40 years or older [4]. It mainly affects the motor system at the substantia nigra. It causes tremors, bradykinesia, rigidity, and other non-motor dysfunctions, such as cognitive and mood disorders. However, the exact pathogenesis of both AMD and PD remains unclear. It is worth noting that they share some risk factors in common, such as aging, but contain some other factors leading different direction of effect on them, such as sex and smoking [5–7]. Though debatable, some researchers do classify both of them as a neurodegenerative disorder [5]. Besides, as the worldwide life expectancy extends, there is an increasing concern for these diseases. Therefore, further studies are important to understand the relationship between AMD and PD.

Several studies have reported a higher tendency for AMD patients to have PD [8, 9]. However, some of them did not adjust for other comorbidities which are often seen in the elderly, and for which Choi et al. [10] did adjust. Unlike previous studies, we aimed to include adequate adjustments to comorbidities and observe their relationship with PD. The purpose of this study was to further evaluate whether there is an increased risk of PD among AMD patients over a longer period, with a more precise subgroup analysis with adjustment for several comorbidities that are often seen among the elderly.

## Methods

### Data source

Taiwan’s National Health Insurance Research Database (NHIRD) was the source of the data used in this study. In addition, National Health Insurance (NHI) program, which was initiated in 1995 and covered 99.9% of Taiwan’s population of 23 million as of 2013, is the national medical insurance and healthcare system of Taiwan. NHIRD includes the data (such as demographic data, medical records, prescription records, and medical procedures) of those who sought medical care through the NHI program. More specifically, this study utilized data from the Longitudinal Health Insurance Database 2000 (LHID2000), which is a sub-database of the NHIRD and is consisted of 1 million people randomly selected from the NHIRD in 2000 [11–13].

### Sample participant

The study population was selected from LHID2000 data covering the period from January 1996 through December 2013. The clinical diagnoses included in the study were determined by licensed physicians and were made based on the International Classification of Diseases, 9th revision, Clinical Modification (ICD-9-CM) Patients newly diagnosed with AMD (ICD-9-CM code: 362.50 Unspecified AMD, 362.51 Non-neovascular AMD, and 362.52 Neovascular AMD) between January 1998 and December 2012 were grouped into the AMD cohort. The date of diagnosis of AMD was used as the index date. Dates between 1998 and 2013 were allocated to the subjects in the non-AMD cohort as index dates. The index date for the non-AMD cohort was determined as follows: (1) Randomly assigning a month number for all people in the non-AMD cohort; (2) Giving them a numeric constant of 15 for the date of the month; (3) Alloting the year 1998 to all people in the non-AMD cohort, calculating their ages, and matching the strings of the calculated ages, gender, and year in the non-AMD cohort to the strings of the ages, gender, and year in the AMD cohort using 1:1 matching; (4) Excluding the matched people from the people in the non-AMD cohort; (5) Repeat the steps 3 and 4 using the year 1999, 2000, 2001, and so on until the year 2012; (6) Finally, each in the AMD cohort was matched with a person in the non-AMD cohort when using 1:1 matching. Starting with the index date, all subjects were followed up until the diagnosis of PD (ICD-9-CM code: 332 and A221), death, withdrawal from the insurance program, or the end of the study period on December 31, 2013. The exclusion criteria for the study were as follows: (1) patients aged between 0 and 49 years old since the index date; (2) patients with PD prior to the index dates. Ultimately, 10,424 AMD patients were enrolled in the AMD group. Subjects in the non-AMD cohort were randomly matched to the patients in the AMD cohort on the basis of age, sex, and index year of AMD diagnosis at a ratio of 1:1. A total of 10,424 subjects were matched.

### Comorbidity and medication

The comorbidities analyzed in this study were osteoporosis (ICD-9-CM code: 733), diabetes (ICD-9-CM code: 250 and A181), cirrhosis (ICD-9-CM code: 571 and A347), cerebrovascular disease (ICD-9-CM code: 362.34 and 430–438), chronic kidney disease (ICD-9-CM code: 403.11, 403.91, 404.12, 404.92, 585, 586, V42.0, V45.1, V56.0, and V56.8), hypertension (ICD-9-CM code: 401–405, A260, and A269), hyperlipidemia (ICD-9-CM code: 272), coronary artery disease (ICD-9-CM code: 410–414), and chronic obstructive pulmonary disease (ICD-9-CM code: 491, 492, 493, and 496). These common comorbidities were identified when diagnosed prior to the index date. The medications analyzed in this study were statin and calcium channel blocker (CCB) [14, 15]. These medications were identified when prescribed prior to the index date.

### Statistical analysis

The chi-square test was used to compare categorical variables between AMD and non-AMD cohorts, whereas the Wilcoxon rank-sum test was used to compare continuous variables between the two cohorts We assessed the overall and age-specific, sex-specific, comorbidity-specific, and medication-specific incidences of PD between the two cohorts. Univariate Cox proportional hazards regression models were used to assess the risk of PD expressed by the hazard ratios (HR) and the 95% confidence intervals (CI). A multivariate Cox proportional hazards regression model was performed with variables, including AMD, sex, age, comorbidities of osteoporosis, diabetes, cirrhosis, cerebrovascular disease, chronic kidney disease, hypertension, hyperlipidemia, coronary artery disease, chronic obstructive pulmonary disease, and medications of statin and CCB. Cumulative incidence rates of PD in the two cohorts were estimated using Kaplan-Meier methods, and the difference of the cumulative incidence curves of PD between the two cohorts was examined by the log-rank test. Analyses were performed using SAS software (version 9.4 for windows; SAS Institute, Cary, NC, USA) for Windows 10. All statistical significance was set at a *p* < 0.05.

## Results

A total of 20,848 patients were enrolled in the present study. Table 1 demonstrates the distributions of sex, age, comorbidities, and medications between the two cohorts. Both cohorts had similar distributions of age and sex. The mean age (± SD) of the study subjects was 70.4 ± 9.6 years in the AMD cohort and 70.3 ± 9.6 years in the non-AMD cohort (Wilcoxon rank-sum test, *p* = 0.27). The AMD patients had higher prevalence of all comorbidities than subjects in the non-AMD cohort (*p* < 0.0001). There was a slightly different drug usage status (statin and CCB) between the two cohorts (*p* < 0.0001). The follow-up time was also different between the two cohorts (Wilcoxon rank-sum test, *p* = 0.0002).

**Table 1:** Baseline characteristics of patients

	Age-related Macular Degeneration (***n*** = 10,424)		Non - Age-related Macular Degeneration (***n*** = 10,424)		***P***-value*
	n	%	N	%	
**Sex**					> 0.99
Female	5130	49.2	5130	49.2	
Male	5294	50.8	5294	50.8	
**Age, years**					> 0.99
50–59	1732	16.6	1732	16.6	
60–69	3087	29.6	3087	29.6	
70–79	3841	36.9	3841	36.9	
80–100	1764	16.9	1764	16.9	
Mean (SD)^b^	70.4 (9.55)		70.3 (9.63)		0.27
**Comorbidity**					
Osteoporosis	2596	24.9	2017	19.4	< 0.0001
Diabetes^a^	4918	47.2	3774	36.2	< 0.0001
Cirrhosis	4060	38.9	3092	29.7	< 0.0001
Cerebrovascular disease	2720	26.1	2226	21.3	< 0.0001
Chronic kidney disease	715	6.86	540	5.18	< 0.0001
Hypertension	7418	71.2	6507	62.4	< 0.0001
Hyperlipidemia	4644	44.5	3450	33.1	< 0.0001
Coronary artery disease	792	7.60	589	5.65	< 0.0001
Chronic obstructive pulmonary disease	4238	40.7	3527	33.8	< 0.0001
Numbers of comorbidity					< 0.0001
0	904	8.67	1863	17.87	
≥ 1	9520	91.33	8561	82.13	
**Medications**					
Statin	8619	82.7	8844	84.8	< 0.0001
CCB	8713	83.6	8683	83.3	< 0.0001
**Follow-up time, year** (SD)^b^	5.66 (3.81)		5.48 (3.84)		0.0002

*Abbreviation*s: *CCB* Calcium Channel Blocker, *SD* Standard Deviation

*P-value using *chi-square* for the comparisons between with and without age-related macular degeneration

^a^Diabetes mellitus included type 1 and type 2 diabetes mellitus

^b^Average age and follow-up time using *Wilcoxon rank-sum test* for verification

Table 2 presents the results of PD risk associated with AMD. AMD patients exhibited a significantly higher risk of subsequent PD, with a crude HR of 1.52 (95% CI = 1.31–1.77). After adjusting all the related covariates, the multivariate Cox proportional hazards regression model indicated an adjusted HR (aHR) of 1.35 (95% CI = 1.15–1.57) for AMD patients compared to the non-AMD cohort. Compared to female patients, male patients had a significant 1.26- (95% CI = 1.07–1.48) fold higher adjusted hazard for PD. Compared to the 50–59 years old age group, the group with 60–69, 70–79, and 80–100 years old subjects had a significant 2.88- (95% CI = 1.91–4.34), 5.12- (95% CI = 3.44–7.62), and 6.12- (95% CI = 4.02–9.31) fold higher adjusted hazard for PD, respectively. Except patients with hyperlipidemia might reduce the risk of PD (aHR = 0.82, 95% CI = 0.69–0.97), patients with diabetes (aHR = 1.21, 95% CI = 1.03–1.42), cerebrovascular disease (aHR = 1.60, 95% CI = 1.36–1.89), and hypertension (aHR = 1.55, 95% CI = 1.27–1.89) could increase the risk of PD. Compared to patients without comorbidity, the aHR of PD was 2.43- (95% CI = 1.76–3.36) fold higher in those with at least one comorbidity. For patients treated with CCB, they were at lower risk of PD.

**Table 2:** Cox model measured hazard ratios and 95% confidence interval of Parkinson’s disease associated with age-related macular degeneration, sex, age, comorbidity, numbers of comorbidity, and medication

Variable	Parkinson’s disease			Crude HR (95% CI)	Adjusted HR (95% CI)
	Event	PY	IR		
**Age-related Macular Degeneration**					
No	275	57,168	4.81	**1(reference)**	**1(reference)**
Yes	433	59,008	7.34	1.52 (1.31–1.77)***	1.35 (1.15–1.57)***
**Sex**					
Female	306	58,550	5.23	**1(reference)**	**1(reference)**
Male	402	57,625	6.98	1.34 (1.15–1.55)***	1.26 (1.07–1.48)**
**Age, years**					
50–59	27	21,129	1.28	**1(reference)**	**1(reference)**
60–69	160	37,650	4.25	3.32 (2.21–4.99)***	2.88 (1.91–4.34)***
70–79	364	42,840	8.50	6.66 (4.50–9.85)***	5.12 (3.44–7.62)***
80–100	157	14,556	10.8	8.57 (5.69–12.9)***	6.12 (4.02–9.31)***
*P* for trend				< 0.0001	< 0.0001
**Comorbidity**					
Osteoporosis					
No	526	92,789	5.67	**1(reference)**	**1(reference)**
Yes	182	23,387	7.78	1.37 (1.16–1.62)***	1.18 (0.98–1.41)
Diabetes					
No	377	72,100	5.23	**1(reference)**	**1(reference)**
Yes	331	44,075	7.51	1.44 (1.24–1.67)***	1.21 (1.03–1.42)*
Cirrhosis					
No	445	78,977	5.63	**1(reference)**	**1(reference)**
Yes	263	37,198	7.07	1.25 (1.08–1.46)**	1.15 (0.98–1.35)
Cerebrovascular disease					
No	457	93,109	4.91	**1(reference)**	**1(reference)**
Yes	251	23,066	10.88	2.23 (1.91–2.60)***	1.60 (1.36–1.89)***
Chronic kidney disease					
No	663	111,329	5.96	**1(reference)**	**1(reference)**
Yes	45	4847	9.28	1.57 (1.16–2.12)**	1.09 (0.80–1.48)
Hypertension					
No	145	42,494	3.41	**1(reference)**	**1(reference)**
Yes	563	73,682	7.64	2.24 (1.87–2.70)***	1.55 (1.27–1.89)***
Hyperlipidemia					
No	466	77,239	6.03	**1(reference)**	**1(reference)**
Yes	242	38,936	6.22	1.03 (0.88–1.20)	0.82 (0.69–0.97)*
Coronary artery disease					
No	649	109,614	5.92	**1(reference)**	**1(reference)**
Yes	59	6561	8.99	1.52 (1.16–1.98)**	1.01 (0.77–1.33)
Chronic obstructive pulmonary disease					
No	399	77,468	5.15	**1(reference)**	**1(reference)**
Yes	309	38,707	7.98	1.55 (1.34–1.80)***	1.07 (0.91–1.25)
**Numbers of comorbidity**					
0	41	18,974	2.16	**1(reference)**	**1(reference)**
≥ 1	667	97,201	6.86	3.19 (2.32–4.37)***	2.43 (1.76–3.36)***
**Medication**					
Statin					
No	168	26,943	6.24	**1(reference)**	**1(reference)**
Yes	540	89,232	6.05	0.97 (0.81–1.16)	0.86 (0.72–1.03)
CCB					
No	172	28,424	6.05	**1(reference)**	**1(reference)**
Yes	536	87,751	6.11	1.01 (0.85–1.20)	0.77 (0.64–0.93)**

*Abbreviations*: *aHR* Adjusted Hazard Ratio estimated by the model including the variables of age-related macular degeneration, sex, age, osteoporosis, diabetes, cirrhosis, cerebrovascular disease, chronic kidney disease, hypertension, hyperlipidemia, coronary artery disease, chronic obstructive pulmonary disease, statin, and CCB, *CCB* Calcium Channel Blocker, *CI* Confidence Interval, *HR* Hazard Ratio, *IR* Incidence Rate, per 1000 person-years, *PY* Person-Years

*p < 0.05; **p < 0.01; ***p < 0.001

Table 3 presents the sex-, age-, comorbidity-, and medication-specific stratified analyses. When stratified by sex, the risk of PD in the AMD cohort was higher in both female (aHR = 1.42, 95% CI = 1.13–1.80) and male patients (aHR = 1.28, 95% CI = 1.05–1.57) compared to the non-AMD cohort. The incidence rate (IR) of PD increased with age in both cohorts, and AMD patients had a significantly higher risk of PD than non-AMD patients in 60–69 (aHR = 1.51, 95% CI = 1.09–2.09), 70–79 (aHR = 1.30, 95% CI = 1.05–1.60), and 80–100 (aHR = 1.40, 95% CI = 1.01–1.95) subgroups. A significant association between increased risk of PD and AMD was observed among patients with comorbidities of osteoporosis (aHR = 1.68, 95% CI = 1.22–2.33), diabetes (aHR = 1.41, 95% CI = 1.12–1.78) and hypertension (aHR = 1.36, 95% CI = 1.15–1.62) and medications of statin (aHR = 1.42, 95% CI = 1.19–1.69) and CCB (aHR = 1.32, 95% CI = 1.11–1.58).

**Table 3:** Incidence rate and hazard ratio of PD between with and without age-related macular degeneration stratified by sex, age, comorbidity, numbers of comorbidity, and medication

Variable	Age-related Macular Degeneration						Crude HR (95% CI)	Adjusted HR (95% CI)
	No			Yes				
	Event	PY	IR	Event	PY	IR		
**Sex**								
Female	115	28,927	3.98	191	29,623	6.45	1.62 (1.29–2.04)***	1.42 (1.13–1.80)**
Male	160	28,240	5.67	242	29,385	8.24	1.45 (1.19–1.78)***	1.28 (1.05–1.57)*
**Age, years**								
50–59	9	10,684	0.84	18	10,445	1.72	2.05 (0.92–4.56)	1.31 (0.57–3.01)
60–69	60	18,836	3.19	100	18,814	5.32	1.67 (1.21–2.30)**	1.51 (1.09–2.09)*
70–79	148	20,681	7.16	216	22,159	9.75	1.36 (1.10–1.68)**	1.30 (1.05–1.60)*
80–100	58	6967	8.32	99	7589	13.0	1.57 (1.14–2.17)**	1.40 (1.01–1.95)*
**Comorbidity**								
Osteoporosis	51	9755	5.23	131	13,631	9.61	1.82 (1.32–2.52)***	1.68 (1.22–2.33)**
Diabetes	109	18,056	6.04	222	26,020	8.53	1.41 (1.12–1.78)**	1.41 (1.12–1.78)**
Cirrhosis	94	15,195	6.19	169	22,003	7.68	1.25 (0.97–1.60)	1.20 (0.93–1.54)
Cerebrovascular disease	92	9742	9.44	159	13,324	11.93	1.27 (0.98–1.64)	1.25 (0.96–1.62)
Chronic kidney disease	18	1942	9.27	27	2904	9.30	1.02 (0.56–1.85)	0.99 (0.54–1.81)
Hypertension	206	33,161	6.21	357	40,520	8.81	1.41 (1.19–1.68)***	1.36 (1.15–1.62)***
Hyperlipidemia	86	15,963	5.39	156	22,973	6.79	1.27 (0.97–1.65)	1.17 (0.90–1.52)
Coronary artery disease	23	2596	8.86	36	3965	9.08	1.02 (0.60–1.71)	1.01 (0.59–1.70)
Chronic obstructive pulmonary disease	117	16,690	7.01	192	22,017	8.72	1.25 (0.99–1.57)	1.19 (0.94–1.50)
**Numbers of comorbidity**								
0	28	12,790	2.19	13	6184	2.10	0.95 (0.49–1.84)	0.95 (0.49–1.85)
≥ 1	247	44,378	5.57	420	52,823	7.95	1.43 (1.22–1.67)*	1.40 (1.20–1.64)***
**Medication**								
Statin	208	44,636	4.66	332	44,596	7.44	1.60 (1.34–1.90)***	1.42 (1.19–1.69)***
CCB	207	42,835	4.83	329	44,916	7.32	1.52 (1.27–1.80)***	1.32 (1.11–1.58)**

*Abbreviations*: *aHR* Adjusted Hazard Ratio estimated by the model including the variables of age-related macular degeneration, sex, age, osteoporosis, diabetes, cirrhosis, cerebrovascular disease, chronic kidney disease, hypertension, hyperlipidemia, coronary artery disease, chronic obstructive pulmonary disease, statin, and CCB, *CCB* Calcium Channel Blocker, *CI* Confidence Interval, *HR* Hazard Ratio, *IR* Incidence Rate, per 1000 person-years, *PY* Person-Years

*p < 0.05; **p < 0.01; ***p < 0.001

The association between different types of AMD and risk of PD was further analyzed. Table 4 shows that AMD patients with ICD-9-CM code: 362.50 (Unspecified AMD) had a significantly higher risk of PD (aHR = 1.33, 95% CI = 1.14–1.54) in relation to the non-AMD cohort.

**Table 4:** Hazard ratio of Parkinson’s disease associated with different types of age-related macular degeneration

Variable	Parkinson’s disease			Crude HR (95%CI)	Adjusted HR (95%CI)
	Event	PY	IR		
**Type of Age-related Macular Degeneration**					
**Unspecified AMD (ICD-9-CM code: 362.50)**					
No	326	64,236	5.08	**1(reference)**	**1(reference)**
Yes	382	51,939	7.35	1.45 (1.25–1.68)***	1.33 (1.14–1.54)***
**Non-neovascular AMD (ICD-9-CM code: 362.51)**					
No	634	107,589	5.89	**1(reference)**	**1(reference)**
Yes	74	8587	8.62	1.46 (1.15–1.86)**	1.19 (0.94–1.52)
**Neovascular AMD** (**ICD-9-CM code: 362.52)**					
No	666	110,575	6.02	**1(reference)**	**1(reference)**
Yes	42	5600	7.50	1.25 (0.91–1.70)	1.09 (0.80–1.49)

*Abbreviations*: *aHR* Adjusted Hazard Ratio estimated by the model including the variables of age-related macular degeneration, sex, age, osteoporosis, diabetes, cirrhosis, cerebrovascular disease, chronic kidney disease, hypertension, hyperlipidemia, coronary artery disease, chronic obstructive pulmonary disease, statin, and CCB, *CCB* Calcium Channel Blocker, *CI* Confidence Interval, *HR* Hazard Ratio, *IR* Incidence Rate, per 1000 person-years, *PY* Person-Years

*p < 0.05; **p < 0.01; ***p < 0.001

**Fig. 1:**
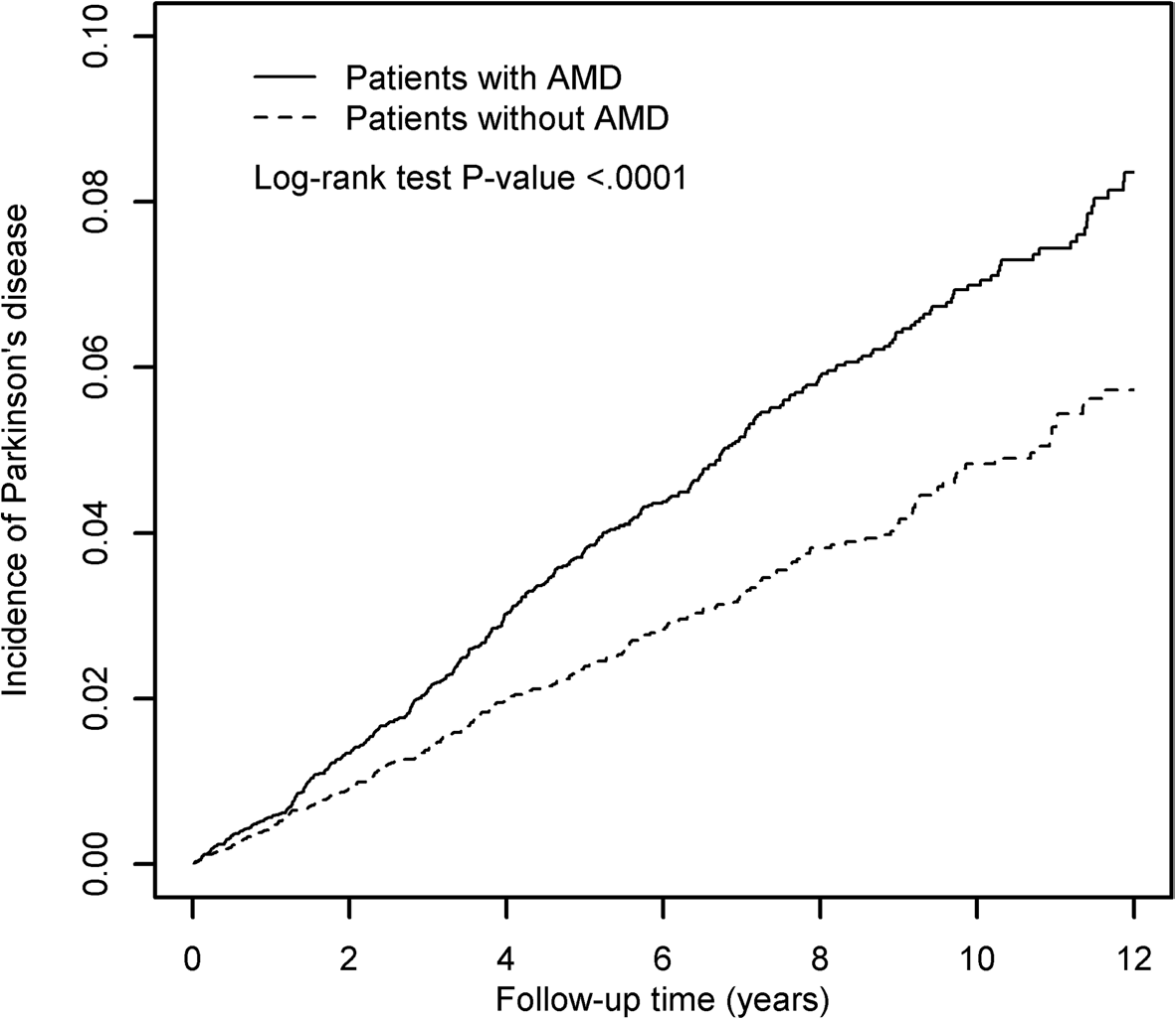
Using Kaplan-Meier survival statistics, it showed crude overall survival curves by with and without age-related macular degeneration. (log-rank test *P* < 0.0001)

Figure 1 demonstrates that the AMD cohort had a higher cumulative incidence of PD than the non-AMD cohort at the end of follow-up (log-rank test, *p* < 0.0001).

## Discussion

Various mechanisms have supported several common conditions linking AMD to PD. First, both diseases are associated with inflammation. For example, a high concentration of complement factors was found in AMD in some studies [16]. Others also supported the role of inflammation and neurodegeneration in AMD and PD [17–19]. Second, autophagy dysfunction results in the accumulation of misfolded proteins. This is reflected by the aggregation of lipofuscin in RPE cells of AMD patients [20] and alpha-synuclein in neurons of PD patients [21]. Third, both the inflammatory process and autophagy dysfunction will induce oxidative stress, which is another common condition found in AMD and PD as both retina cells and brain cells consume a high proportion of oxygen [22, 23]. When the function of relieving oxidative stress declines due to aging and mitochondrial dysfunction in the RPE of AMD patients and the brain of PD patients, levels of reactive oxygen species and oxidative damage increase.

Structurally, both AMD and PD patients have been reported to undergo thinning of the RNFL [24–26]. Furthermore, this thinning process was related to the duration of PD. The phenomenon was reported to be due to the loss of retinal dopaminergic amacrine cells and impairment of ganglion cell axons. Based on this finding, the PD rating scale was made to predict the severity of PD based on the thickness of RNFL and other retinal layers by optical coherence tomography [27]. Among these studies, one study reported a different result [28], where there was no difference in retinal thickness between the PD patients and the control group, even though visual acuity and contrast sensitivity were impaired. This could be attributed to the older age of this study group compared to other studies. The average age difference could lead to more difficulties in detecting the different thicknesses of RNFL and the other parts of the retina, since age itself is also a negative factor. Other related studies indicated the potential association between AMD and PD, such as the use of L-DOPA in treating PD that was reported as having a role in preventing AMD [29]. In addition, a lower cognitive function was found among AMD patients [30, 31].

Several studies examined the association between AMD and PD, and all concluded that AMD patients have a higher risk of PD. In 2014, Chung et al. found that neovascular AMD is highly associated with PD during a 3-year follow-up period [8]. They identified 877 cases of neovascular AMD and over 8770 controls between January 1, 2001 and December 31, 2008 in a 3-year follow-up period adjusted for monthly income, geographic region, hypertension, diabetes, hyperlipidemia, and coronary heart disease. All subjects were older than 40 years. The diagnosis of neovascular AMD included ICD-9-CM code 362.42 (serous detachment of RPE), 362.43 (serous detachment of RPE), 362.52 (exudative AMD), and 362.53 (Cystoid macular degeneration). In 2018, Etminan et al. suggested that neovascular AMD may predict the onset of PD [9]. They used the Canadian British Columbia Retinal Disease Database from 2009 to 2013. Neovascular AMD patients undergoing intravitreal injections (bevacizumab or ranibizumab) were included, but only adjusted for age in the cohort study. In 2019, the most updated publication by Choi et al. elucidated that AMD was associated with higher PD risk [10]. They determined 2213 AMD and 306,127 non-AMD follow-up from 2006 to 2013, with adjustment for age, sex, household income, smoking, alcohol, physical activity, BMI, SBP, fasting glucose, total cholesterol, and Charlson comorbidity index. The study defined AMD by ICD-10 coded as H35.3 without specifying neovascular or non-neovascular AMD. Participants were all over 50 years. These three studies above adjusted their lifestyle, socioeconomic status, and clinical conditions.

Our result among non-neovascular and neovascular AMD was different from that of Chung and Etminan [8, 9], but consistent to Choi’s study [10]. The reason might because that our study involved AMD with adjustment for different comorbidities and medications which is similar to Choi et al. [10]. Since the detachment of RPE or cystoid macular edema could be two of clinical features of neovascular AMD but not exclusive ones. Thus, unlike Chung et al. [8], we defined AMD, including unspecific, non-neovascular and neovascular types, as ICD-9-CM codes 362.50, 362.51, and 362.52 rather than as the codes of AMD’s clinical features. In addition, participants younger than 50 years were excluded from our study. In addition, a relatively large number of AMD cases with long follow-up period also offered a more comprehensive result. These may make our result different from that of Chung and Etminan [8, 9], but consistent to Choi study [10].

During the analysis of AMD subtypes (Unspecified, non-neovascular, and neovascular AMD), there was no significant difference in risk of PD. The non-significant difference of PD risk between non-neovascular and neovascular may need further researches stratifying by the disease’s stage since it does not define the stage of AMD by the ICD-9-CM codes as non-neovascular or neovascular AMD. The follow up time in our study started from that AMD was diagnosed rather than the true duration of the disease which might be even longer than the time we recorded. Perhaps the duration or the stage of the AMD might be associated with different degree of the risk of PD.

One of the strengths of our study was the adjustment of sufficient clinical comorbidities and medications related to aging, with a large amount of data that could offer a statistically meaningful outcome. Although we did not adjust the lifestyle, which was proven to be a confounder in the model, comorbidities such as hypertension and hyperlipidemia might also reflect the lifestyle. In addition, the number of AMD patients in our study was relatively large, and the follow-up year was relatively long (5.66 ± 3.81 years in the AMD cohort and 5.48 ± 3.84 years in the non-AMD cohort, from 1998 to 2013). However, there were still limitations to this study. First, the inclusion of cases was based on the data from NHIRD. It was not as accurate as the diagnosis based on diagnostic criteria by researchers themselves. Second, the NHIRD contained mostly Taiwanese patients. Therefore, the result is difficult to generalize globally. Third, it was difficult to separate patients with neovascular and non-neovascular AMD since doctors might only classify them with the general term (ICD-9-CM code 362.50) without specifying the type. This made it impossible to determine the significant association between either type of AMD to the risk of PD due to limited specific data (Table 4). The results were consistent with those of Choi et al., who also compared AMD without specifying the subtype [10]. Fourth, there are other degenerative ocular diseases, such as glaucoma, which are also worth surveying. Further studies are suggested to solve these problems.

In conclusion, we have a result that AMD is associated with a higher risk of PD with adjustment for sufficient clinical comorbidities and long follow-up time. However, further studies for comparing the association between PD and neovascular or non-neovascular AMD during long-term follow-up is needed with more information such as the stage of the disease.

## Data Availability

All relevant data are within the manuscript.
